# P-1750. Invasive Fungal Infections in Acute on Chronic Liver Failure Patients at a Tertiary Care Centre in India

**DOI:** 10.1093/ofid/ofaf695.1921

**Published:** 2026-01-11

**Authors:** Jayasree Subramanian, Gagandeep Singh, Immaculata Xess, Manish Soneja, Shalimar

**Affiliations:** All India Institute of Medical Sciences, New Delhi, New Delhi, Delhi, India; All India Institute of Medical Sciences, New Delhi, New Delhi, Delhi, India; All India Institute of Medical Sciences, New Delhi, New Delhi, Delhi, India; All India Institute of Medical Sciences, New Delhi, Delhi, India; All India Institute of Medical Sciences, New Delhi, Delhi, India

## Abstract

**Background:**

Acute on Chronic Liver Failure (ACLF) patients are more prone to Invasive Fungal Infections (IFIs) in view of their impaired immunity. This study aims to determine the incidence of IFIs in this subset of patients, the utility of beta-d-glucan (BDG) for diagnosis and the in-hospital outcomes of these patients.

**Figure:**
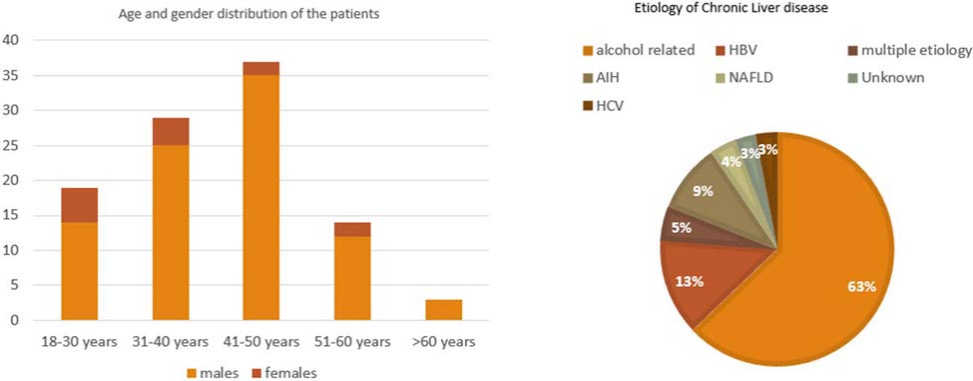

**Figure:**
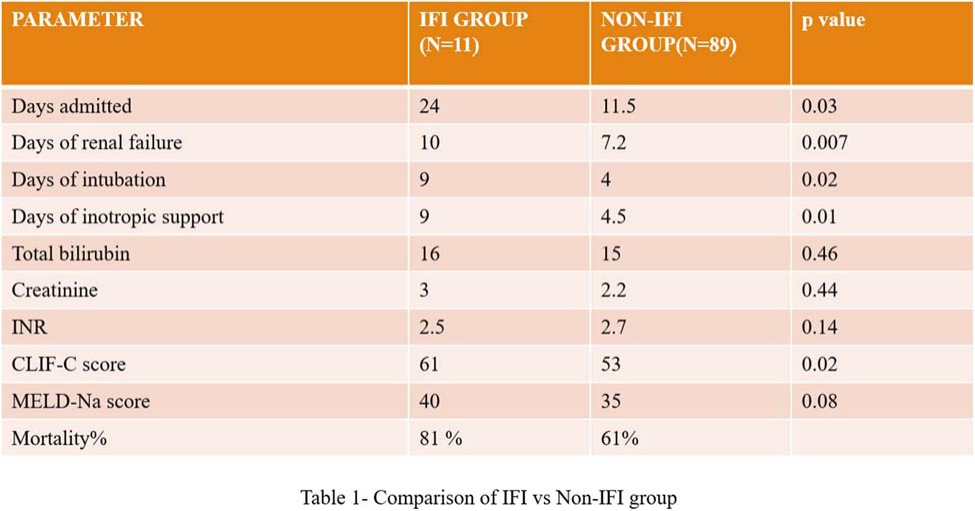

**Methods:**

A prospective observational study was conducted in a tertiary care center from January 2023 till December 2024. Patients with ACLF ≥ 18 years, with evidence of organ/circulatory dysfunction with or without features of infection not responding to antibiotics were enrolled. Demographic, clinical and laboratory parameters were recorded. Blood fungal cultures and serum BDG was collected in these patients. Patients with IFI were diagnosed based on the microbiological, host and clinical factors. These patients were followed up to determine the in-hospital outcome including the length of admission, organ failure and mortality.

**Figure:**
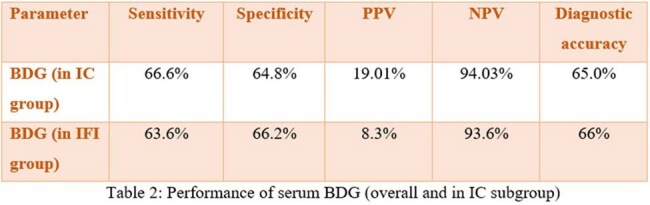

**Figure:**
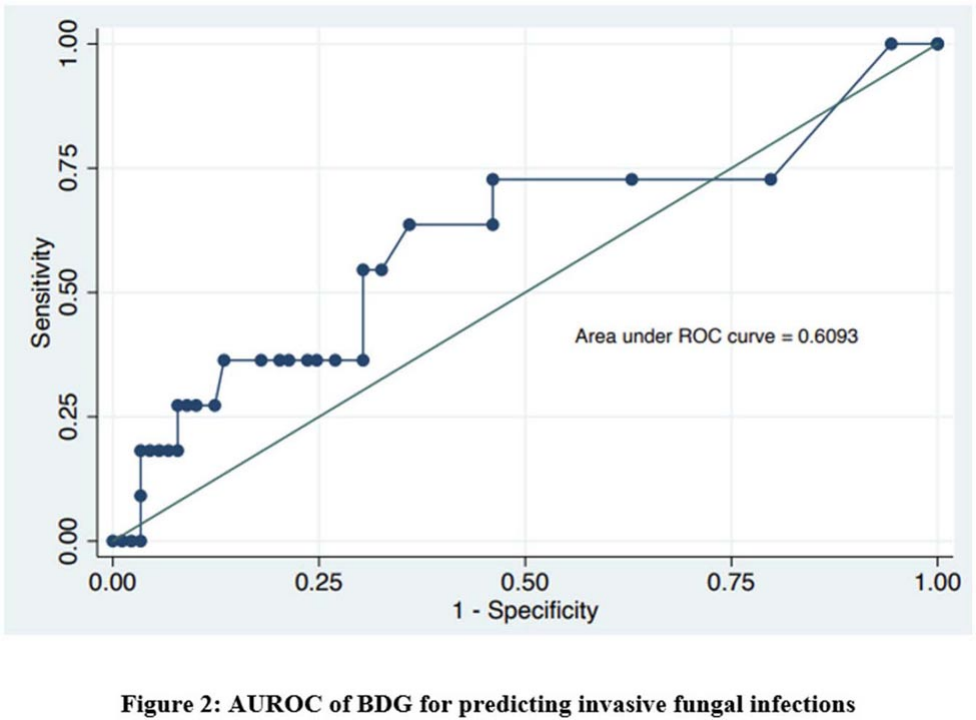

**Results:**

Among the 100 (mean age 40.4 years) patients, 11% of the patients (95% Confidence Interval 5.4% to 19.6%) were found to have IFI. Invasive Candidiasis (IC) was the most common IFI (in 6/11 patients), with *Candida parapsilosis* being the most common pathogen isolated in the blood culture (3/6). Invasive aspergillosis (3/11) was the second most common IFI followed by mucormycosis and cryptococcosis (1/11 each). The sensitivity and specificity of BDG were 63.6 and 66.2% respectively for IFI with a Negative Predictive value (NPV) of 94%. The days of admission, organ failure and inotropic support were significantly higher in patients with IFI. The mortality rate was 81% in the patients with IFI.

**Conclusion:**

IFIs in ACLF patients is associated with increased morbidity and mortality. BDG has moderate specificity and sensitivity but a good NPV in diagnosing IFIs. A high index of clinical suspicion along with mycological parameters are needed for early diagnosis of these infections.

**Disclosures:**

All Authors: No reported disclosures

